# Zika Virus Infection and Its Devastating Consequences

**DOI:** 10.5334/aogh.2601

**Published:** 2019-08-28

**Authors:** Daniel Caplivski

**Affiliations:** 1 Travel Medicine Program, Division of Infectious Diseases, Icahn School of Medicine at Mount Sinai, New York, US

## Abstract

The arrival of Zika virus in the Americas in recent years brought with it a dramatic increase in the rates of microcephaly in regions of Northeastern Brazil. It was from this region of the of the world that we began to understand that this flavivirus (once considered a less severe version of dengue) could have devastating consequences when the infection occurred in pregnant women who did not yet have immunity. We now understand that Zika Congenital Syndrome can have additional effects outside of the central nervous system, but its most lasting consequences are related to the brain infection in utero.

In the four years since the start of the outbreak, we have also come to understand the role of sexual transmission in the spread of Zika virus. It is a trait that has complicated reproductive planning for millions of inhabitants of South America as well as countless other travelers from all over the world. Advances in vaccine development have been encouraging, but a significant research time gap remains before we are likely to have a clinically available vaccine. The rapid onset of the outbreak has been followed by a trailing off of cases to the point that the WHO and CDC modified their recommendations to travelers to the region. Nonetheless, the prevalence of *Aedes egypti* mosquitoes throughout the world make it likely that low-level transmission will continue to occur and many regions will remain at risk for sporadic outbreaks.

Dr. Lima Rocha and colleagues have enlightened our understanding of the epidemiology of this phenomenon in their case-control study from the center of the outbreak. They were able to evaluate 58 cases and 116 controls to further the causal role that Zika infection plays in microcephaly. Their study was completed in the Ceara Region of Brazil, giving us insight into the nature of the outbreak in a region where intense exposure to mosquitos and dense population aided spread of the virus. The authors also examined the potential confounding variables and environmental exposures in confirming that Zika virus infection carried with it a 14-fold increase in the risk of microcephaly among infants born to mothers who were infected during pregnancy. While cases of Zika virus infection are on the wane in the Americas, there remains an important threat of new outbreaks in the future. There current study helps detail the outbreak in its epicenter.

